# Dual role of carcinoembryonic antigen-related cell adhesion molecule 6 expression in predicting the overall survival of gastric cancer patients

**DOI:** 10.1038/s41598-017-11482-9

**Published:** 2017-09-07

**Authors:** Mingde Zang, Lei Hu, Shu Cao, Zhiyuan Fan, Li Pang, Jianfang Li, Liping Su, Chen Li, Wentao Liu, Qinlong Gu, Zhenggang Zhu, Min Yan, Bingya Liu

**Affiliations:** Department of General Surgery, Shanghai Key Laboratory of Gastric Neoplasms, Shanghai Institute of Digestive Surgery, Ruijin Hospital, Shanghai Jiao Tong University School of Medicine, Shanghai, 200025 People’s Republic of China

## Abstract

Carcinoembryonic antigen-related cell adhesion molecule 6 (CEACAM6) is a member of the glycosylphosphatidylinositol-linked immunoglobulin superfamily that is implicated in many human cancers. Here, we aimed to investigate the role of CEACAM6 expression in predicting the overall survival (OS) in gastric cancer (GC). The impact of CEACAM6 on the survival of patients with GC (n = 876) was assessed using an online Kaplan-Meier plotter. Findings were validated using the OS data of patients (*n* = 160) recruited from Ruijin Hospital. We found that high CEACAM6 expression was associated with a better OS in early-stage or well-differentiated GC, or who were treated without 5-fluorouracil (5-FU). Conversely, high CEACAM6 expression was associated with a poor OS in advanced-stage GC, poorly differentiated tumors, or who were treated with 5-FU. Furthermore, CEACAM6 may serve as a better marker for predicting OS in GC than CEA. In addition, CEACAM6 overexpression in GC cells increased apoptotic resistance to 5-FU. Moreover, CEACAM6 induced cluster of differentiation 4- and 8-positive lymphocytes were detected in early-stage GC. In conclusion, CEACAM6 plays a contradictory role in predicting the OS in GC. In early-stage GC, high CEACAM6 expression is associated with improved OS. However, in advanced-stage GC, high CEACAM6 expression is associated with a poor OS.

## Introduction

Gastric cancer (GC) is the fourth most common cancer and the second leading cause of cancer-related deaths worldwide^1, 2^. The health burden of GC in East Asian countries is greater than in other global regions^3–5^. According to the latest cancer statistics, GC is the leading cause of cancer-related deaths in China followed by lung cancer^6^. Due to a lack of validated screening programs, the majority of patients with GC in poor regions and developing countries are diagnosed at an advanced stage, resulting in high mortality^7^. Overall survival (OS) is considered the most important endpoint for evaluating cancer prognosis and is affected by many factors including tumor stage, differentiation, lymph node metastasis, and chemotherapy resistance^8–10^.

Carcinoembryonic antigen-related cell adhesion molecule 5 (CEACAM5/CEA) and CEACAM6 are members of the glycosylphosphatidylinositol-linked immunoglobulin superfamily that are overexpressed in a variety of human cancers^11^, particularly in gastrointestinal cancer^12, 13^ and serve as tumor markers for predicting the prognosis of cancer patients^14, 15^. Furthermore, CEA and CEACAM6 proteins are expressed in immune cells, including neutrophils, T-lymphocytes, and natural killer cells, and are involved in the regulation of monocyte activation^16–19^. CEACAM proteins interact with each other through homophilic and heterophilic intercellular adhesion. Previously, we reported that CEACAM6 was overexpressed in GC tissues and that it promoted migration, invasion, and angiogenesis^20, 21^.

As CEACAM6 is overexpressed in GC, we aimed to assess the relationship between CEACAM6 expression and the OS of patients with GC. In this study, we used Kaplan-Meier plotter to assess the survival of patients with GC^22^. In addition, the OS of patients with GC was further analyzed according to survival data from 160 patients with GC, recruited from Ruijin Hospital (Shanghai, China). Thereafter, OS was analyzed using Kaplan-Meier plots and compared with the results obtained from the online database. Patients were classified into groups depending on tumor stage and differentiation, metastasis, and treatment. We subsequently analyzed the effects of these factors on the OS of patients with GC to better predict prognosis and guide treatment.

## Results

### Role of CEACAM6 expression in predicting the overall survival of patients with gastric cancer

**Figure 1:**
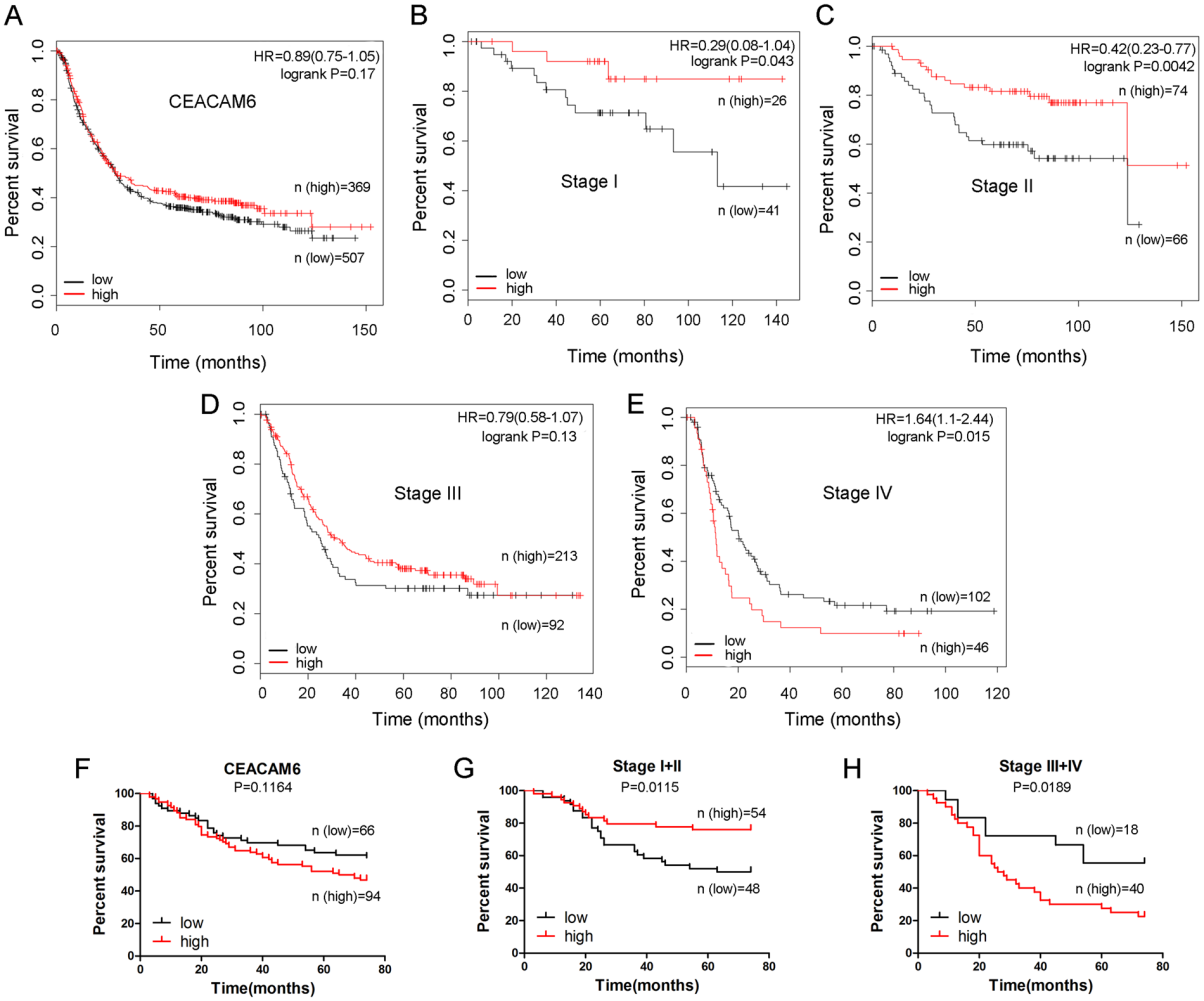
Dual role of CEACAM6 expression in predicting the overall survival of Stage I–II and Stage III–IV gastric cancer. Kaplan-Meier curves of OS according to (**A**) CEACAM6 expression, (**B**) CEACAM6 expression in Stage I GC, (**C**) CEACAM6 expression in Stage II GC, (**D**) CEACAM6 expression in Stage III GC, and (**E**) CEACAM6 expression in Stage IV GC. Kaplan-Meier curves of OS for GC patients (*n = *160) according to (**F**) CEACAM6 staining, (**G**) CEACAM6 staining in early (Stage I–II) GC, and (**H**) CEACAM6 staining in advanced (Stage III–IV) GC.

The prognostic significance of CEACAM6 expression in patients (*n* = 876) with GC was assessed using Kaplan-Meier survival estimates, which revealed that CEACAM6 expression had no effect on the OS of these patients (*P* > 0.05; Fig. 1A and Table 1). However, high CEA expression was found to be associated with a better OS in patients with GC (*P < *0.05; Supplementary Fig. S1A and Table S1). To identify those factors that contribute to these observations, stratified analysis was performed to evaluate the prognostic significance of CEACAM6 and CEA expression in early- and advanced-stage GC. Notably, high CEACAM6 expression was associated with better OS in patients with Stage I–II GC (*P < *0.05 and *P < *0.01, respectively; Fig. 1B–C). No significant association was observed in patients with Stage III GC (*P* > 0.05; Fig. 1D). However, high CEACAM6 expression was associated with a poor OS in patients with Stage IV GC (*P < *0.05; Fig. 1E).

**Table 1:** Association between CEACAM6 expression and overall survival of gastric cancer patients (data from http://kmplot.com/analysis/).

Parameters		Cases	Median Survival(months)	HR(95%CI)	P value
All patients	Low	507	28.1	0.89 (0.75–1.05)	0.17
	High	369	28.7		
I	Low	41	NA	0.29 (0.08–1.04)	**0**.**043**
	High	26	NA		
II	Low	66	28.8	0.42 (0.23 –0.77)	**0**.**0042**
	High	74	123.6		
III	Low	92	25.17	0.79 (0.58–1.07)	0.13
	High	213	33.27		
IV	Low	102	20.3	1.64 (1.1–2.44)	**0**.**015**
	High	46	11.33		
Well differentiation	Low	9	NA	0.59 (0.24–1.47)	0.25
	High	23	NA		
Moderately differentiation	Low	46	56.9	1.96 (1–3.83)	**0**.**045**
	High	21	16.4		
Poorly differentiation	Low	72	33.2	1.82 (1.2–2.76)	**0**.**0042**
	High	93	15.3		
5-Fu chemotherapy	Low	40	18.6	2.1 (1.37–3.23)	**0**.**0005**
	High	113	10.2		
No 5-Fu chemotherapy	Low	35	NA	0.39 (0.16–1)	**0**.**042**
	High	41	NA		

NA: Not Available. HR: Hazard Ratio.

To validate the above findings from the online database, survival data was analyzed for 160 patients who underwent curative resection for GC at Ruijin Hospital (Shanghai, China) (Table 2). No significant difference in OS was observed between patients with high and low CEACAM6 expression (*P* > 0.05; Fig. 1F). Improved OS was observed in Stage I–II patients with high CEACAM6 expression (*P* < 0.05; Fig. 1G). In contrast, high CEACAM6 expression was associated with a poor OS in patients with Stage III–IV GC (*P* < 0.05; Fig. 1H). These findings are comparable to those obtained using the Kaplan-Meier plotter, suggesting high CEACAM6 expression correlates with better OS in patients with early (Stage I–II) GC, but poorer OS in patients with advanced (Stage III–IV) GC (Tables 1 and 2).

**Table 2:** Association between CEACAM6 expression and overall survival of gastric cancer patients (n = 160).

Parameters		Cases	Median Survival(months)	HR(95%CI)	P value
All patients	Low	66	NA	0.6935(0.4391–1.095)	0.1164
	High	94	67.5		
I + II	Low	48	68.5	2.321(1.208–4.461)	**0**.**0115**
	High	54	NA		
III + IV	Low	18	NA	0.4539(0.2348–0.8777)	**0**.**0189**
	High	40	27		
Moderate differentiation	Low	17	NA	0.4469(0.2028–0.9849)	**0**.**0457**
	High	30	29		
Poor differentiation	Low	49	NA	0.5039(0.2994–0.8482)	**0**.**0099**
	High	64	26.5		
Lymph node metastasis	Low	29	56	0.5854(0.3466–0.9888)	**0**.**0453**
	High	58	26.5		
No lymph node metastasis	Low	37	47	1.223(0.6593–2.268)	0.5234
	High	36	62		

NA: Not Available. HR: Hazard Ratio.

### Role of CEACAM6 expression in predicting overall survival in patients with well-, moderately, and poorly differentiated gastric cancer

**Figure 2:**
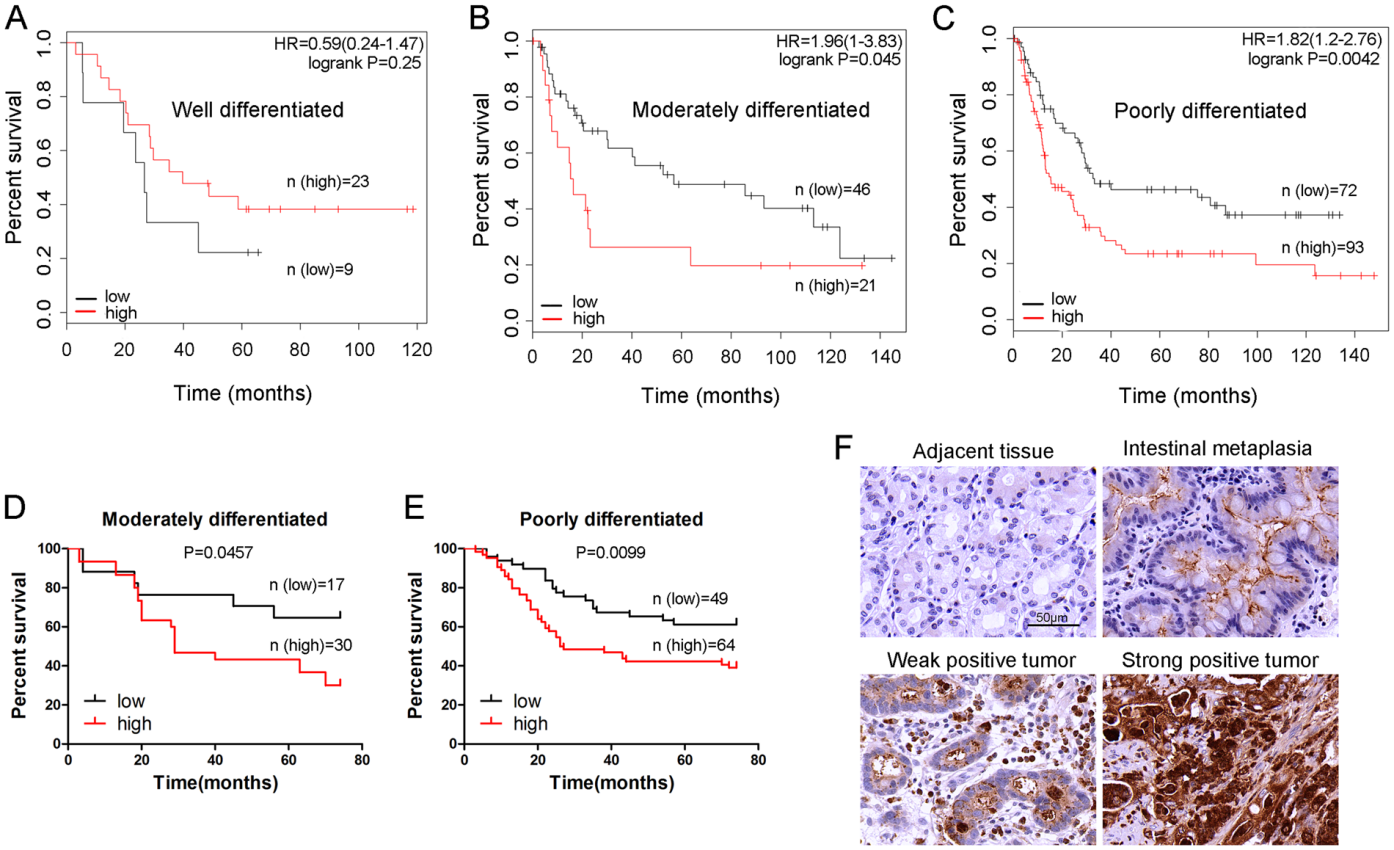
Combined role of CEACAM6 expression and tumor differentiation in predicting the overall survival in patients with gastric cancer. Kaplan-Meier curves of OS according to CEACAM6 expression in (**A**) well-, (**B**) moderately, and (**C**) poorly differentiated GC. Kaplan-Meier curves of OS according to CEACAM6 staining in (**D**) moderately and (**E**) poorly differentiated GC. (**F**) Image of CEACAM6 staining in adjacent tissues, intestinal metaplasia, and tumor tissues (400x magnification).

Survival estimates obtained from the Kaplan-Meier plots were classified according to whether they corresponded to well-, moderately, or poorly differentiated tumors. No significant difference in OS was observed between well-differentiated tumors with high and low CEACAM6 expression (*P* > 0.05; Fig. 2A). However, high CEACAM6 expression was associated with poor OS in patients with moderately or poorly differentiated tumors (*P* < 0.05 and *P* < 0.01, respectively; Fig. 2B,C). Furthermore, a significant difference in OS was observed on CEACAM6 staining between patients with high and low CEACAM6-expressing moderately differentiated tumors (*P* < 0.05; Fig. 2D). High CEACAM6 expression was associated with poor OS in patients with poorly differentiated tumors on CEACAM6 staining (*P* < 0.01; Fig. 2E). Most of the adjacent tissues had negative CEACAM6 staining (Fig. 2F). In some adjacent tissues, intestinal metaplasia was detected with weak CEACAM6 staining. Weak CEACAM6 staining was present in tumors with minor structural disorder, while strong CEACAM6 staining was observed in tumors with major structural disorder, suggesting CEACAM6 disrupts tumor cell polarity and inhibits cell differentiation.

### Role of CEACAM6 expression in predicting overall survival in patients with gastric cancer with and without lymph node metastases

**Figure 3:**
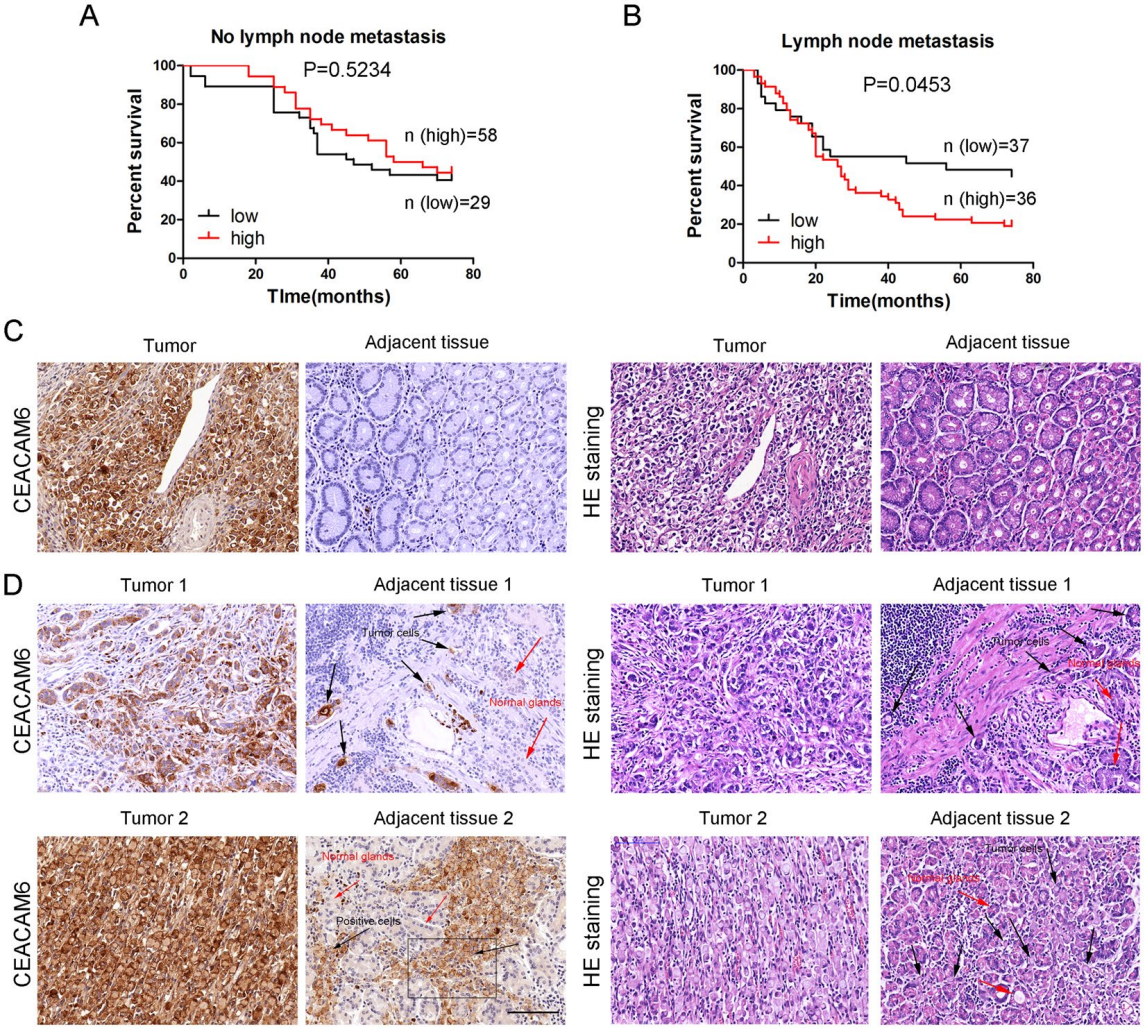
Combined role of CEACAM6 expression and distant metastasis in predicting overall survival in patients with gastric cancer. Kaplan-Meier curves of OS according to CEACAM6 expression in patients (**A**) without and (**B**) with lymph node metastasis. Images of CEACAM6 and HE staining in tumor and adjacent tissues in (**C**) early-stage and (**D**) advanced-stage GC. In panel 1, CEACAM6-positive tumor cells are apparent in the vascular and interstitial space of adjacent tissues. In panel 2, signet ring cancer cells are apparent with strong CEACAM6 staining and tumor cell invasion through the interstitial space and into adjacent tissues (black arrow: CEACAM6-positive tumor cells, red arrow: normal gastric glands).

In patients with GC without lymph node metastasis, no significant difference was observed (*P* > 0.05; Fig. 3A). However, high CEACAM6 expression was associated with poor OS in patients with lymph node metastasis (*P* < 0.01; Fig. 3B). These findings suggest that CEACAM6 contributes to better OS in GC patients without metastasis, but poorer OS in patients with metastasis. In CEACAM6 positive early-stage GC, CEACAM6-positive tumor cells were not detected in adjacent tissues (Fig. 3C). However, most tumor tissues were stained with strong CEACAM6 in advanced-stage GC and CEACAM6-positive tumor cells were detected in vascular and interstitial tissues (Fig. 3D). These observations suggest that CEACAM6-positive tumor cells are more available to migrate into adjacent normal tissues in advanced-stage GC than in early-stage GC, resulting a poor OS.

### Combined role of CEACAM6 expression and chemotherapy with 5-fluorouracil in predicting overall survival in patients with gastric cancer

**Figure 4:**
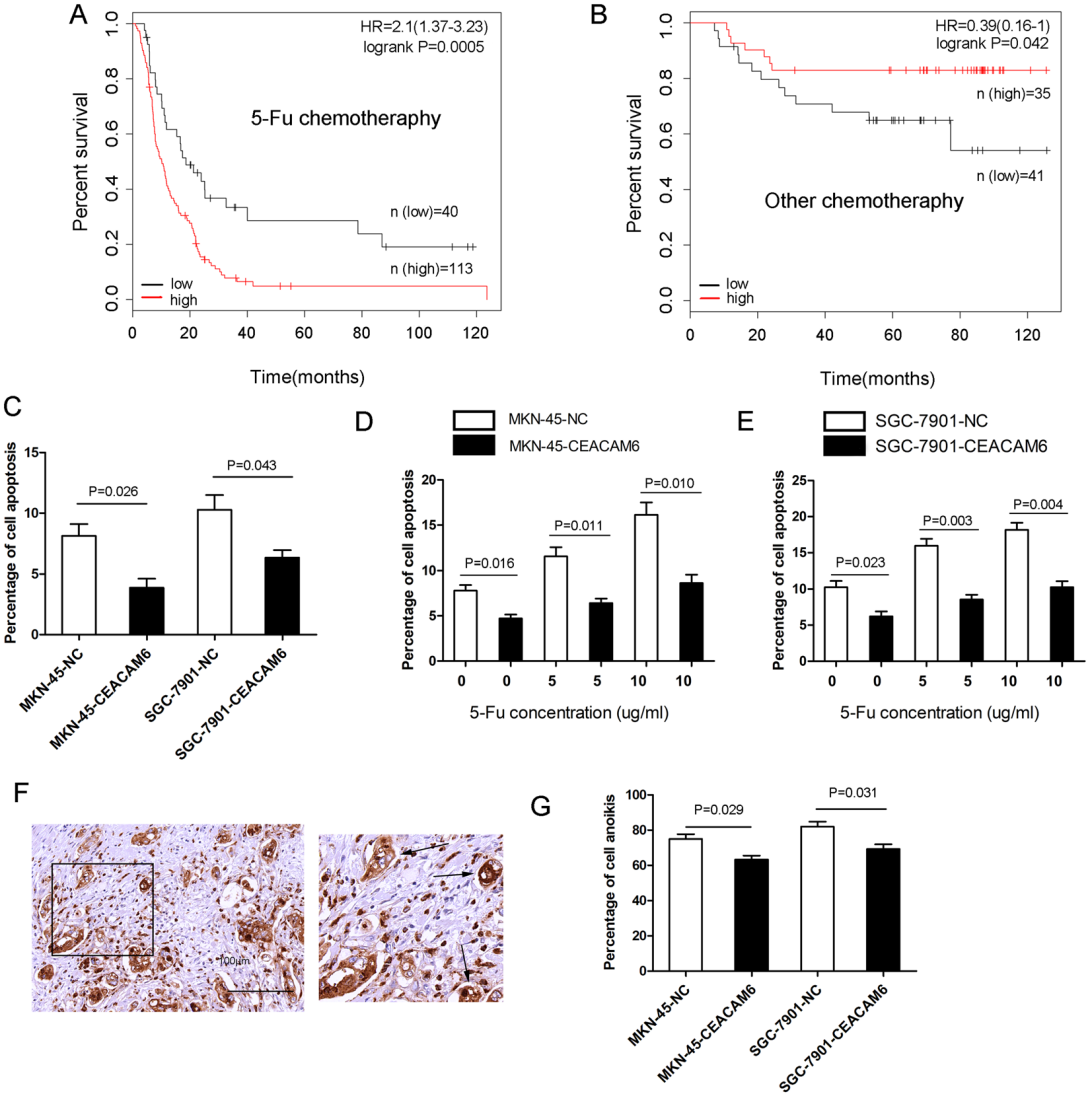
Combined role of CEACAM6 expression and chemotherapy with 5-fluorouracil in predicting the overall survival in patients with gastric cancer. Kaplan-Meier curves of overall survival according to CEACAM6 expression in patients who were treated with adjuvant chemotherapy (**A**) with and (**B**) without 5-FU. (**C**) CEACAM6 overexpression inhibits apoptosis in MKN45 and SGC7901 GC cell lines. (**D**,**E**) CEACAM6 overexpression affects apoptosis in GC cell lines treated with different concentrations of 5-FU (0.0, 5.0, and 10.0 µg/mL). (**F**) Dissociated tumor cells (black arrow) exhibit strong positive staining for CEACAM6. (**G**) CEACAM6 overexpression inhibits anoikis in GC cell lines.

Five-fluorouracil (5-FU) is commonly used in the treatment of GC. However, our observations show that it may not be suitable in certain situations. We found that in patients who received adjuvant chemotherapy with 5-FU, high CEACAM6 and CEA expression were associated with poor OS (*P* < 0.001 and *P* < 0.05, respectively; Fig. 4A and Supplementary Fig. S2A). However, high CEACAM6 and CEA expression were associated with better OS in patients who received adjuvant chemotherapy without 5-FU (*P* < 0.05 and *P* = 0.01, respectively; Fig. 4B and Supplementary Fig. S2B). These findings suggest that patients with high CEACAM6 or CEA expression are not sensitive to 5-FU treatment. To verify this, apoptotic assays were performed. CEACAM6 overexpression increased apoptotic resistance in MKN45 and SGC7901 GC cell lines compared to controls (Fig. 4C). Furthermore, when apoptotic assays were performed after GC cells were exposed to different concentrations of 5-FU (0.0, 5.0, and 10.0 μg/mL), CEACAM6 expression was associated with increased resistance to 5-FU (Fig. 4D,E). In GC tissues, tumor cells that had dissociated from the matrix exhibited strong CEACAM6 staining and formed spheres without apoptosis (Fig. 4F). This observation suggests CEACAM6 also inhibits anoikis in GC, making metastasis more available. This hypothesis was confirmed using an anoikis assay (Fig. 4G).

### Cluster of differentiation 4 and 8 expression in early- and advanced-stage gastric cancer

**Figure 5:**
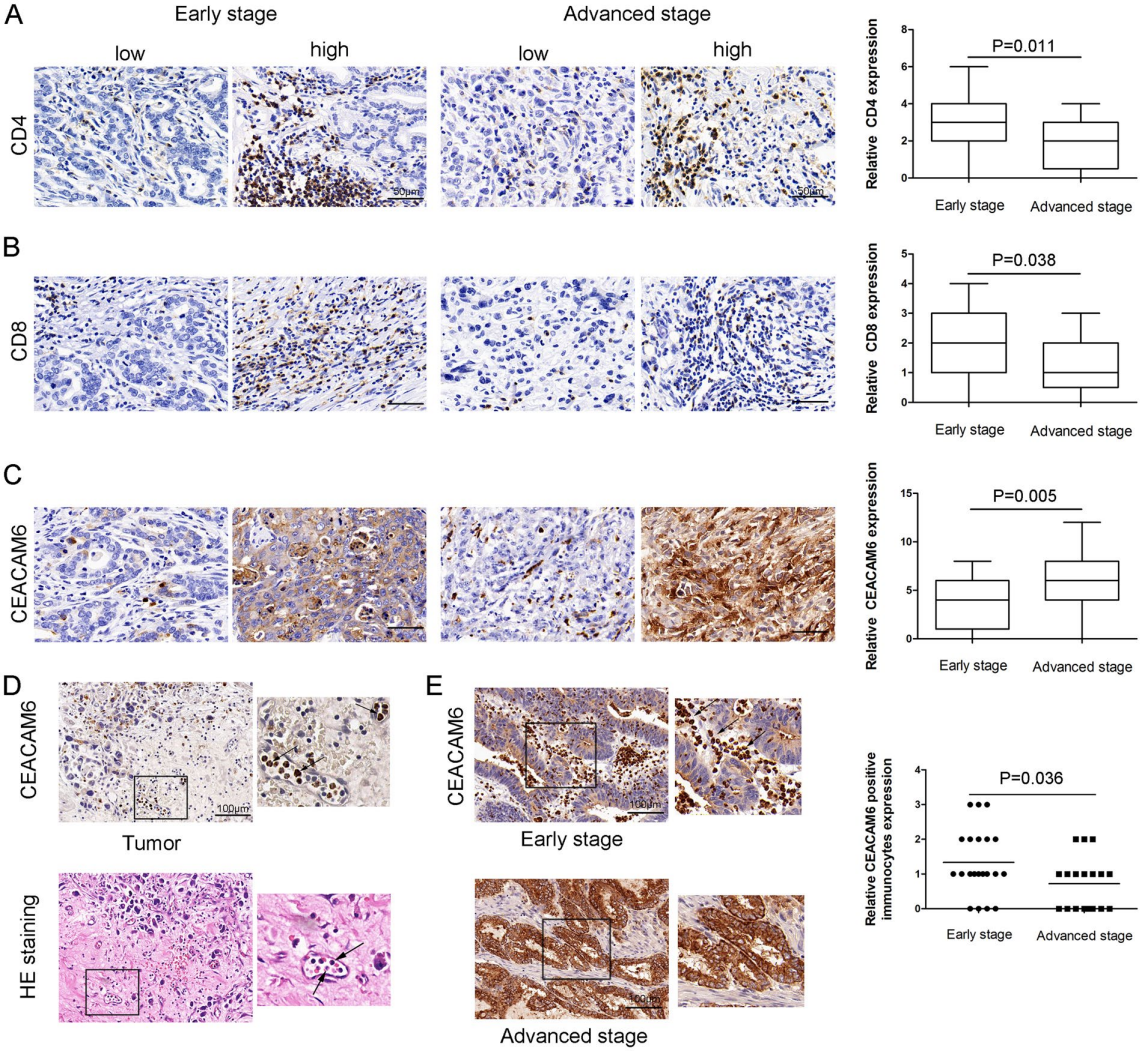
Cluster of differentiation (CD) 4- and CD8-positive expression in early- and advanced-stage gastric cancer. Representative images of (**A**) CD4-positive and (**B**) CD8-positive staining in early- and advanced-stage GC. (**C**) CEACAM6 staining scores in early- and advanced-stage GC. (**D**) CEACAM6-positive immunocytes (black arrow) in the vascular space of the tumor. (**E**) Representative images of immunocytes in early-stage GC tissue (black arrow: CEACAM6-positive immunocytes). Immunocytes were rarely detected in advanced-stage GC.

To better understand the contradictory role of CEACAM6 expression in predicting the OS of patients with GC, early- and advanced-stage GC tissues were stained for cluster of differentiation (CD) 4 and CD8. The results showed that CD4- and CD8-positive cells were more numerous in early-stage GC tissues than in advanced-stage GC tissues (*P* = 0.01 and *P* < 0.05, respectively; Fig. 5A,B). In addition, higher CEACAM6 expression was observed in advanced-stage GC tissues than in early-stage GC tissues (*P* < 0.01; Fig. 5C). CEACAM6-positive immune cells were also detected in vascular tissues (Fig. 5D). A greater number of CEACAM6-positive immune cells were detected in early-stage GC tissues than in advanced-stage GC tissues, where almost none were detected (*P* < 0.05; Fig. 5E).

### Proposed mechanism of CEACAM6 function in early- and advanced-stage gastric cancer

**Figure 6:**
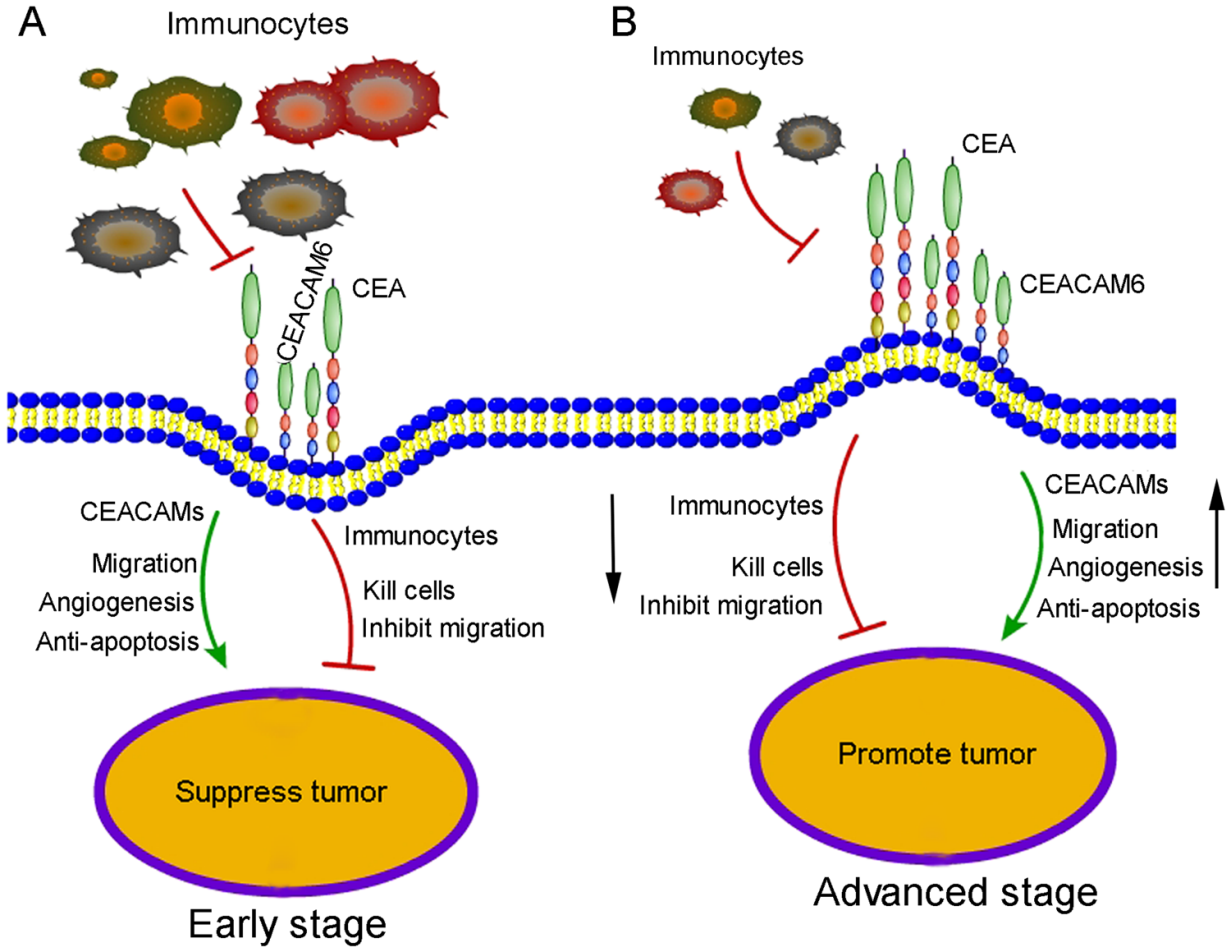
Proposed mechanism for CEACAM6 function in early- and advanced-stage gastric cancer. (**A**) In early-stage GC, immunocytes are abundant and the body’s immune response is very strong. Immunocytes expressing CEACAM6 are more likely to interact with CEACAM6-positive tumor cells through homophilic and heterophilic intercellular interactions. Therefore, the majority of tumor cells are suppressed or killed. Thus, high CEACAM6 expression is associated with better overall survival in patients with early-stage GC. (**B**) Immunocytes were absent in advanced-stage GC. However, CEACAM6 expression is increased, which promotes tumor metastasis, anti-apoptosis, and angiogenesis. Consequently, high CEACAM6 expression is associated with a poor overall survival in patients with advanced-stage GC.

Both promotive and suppressive factors contribute to OS during GC progression, with the final outcome based on their combined effect. In early-stage GC, immunocytes are abundant and the body’s immune response is very strong. Immunocytes express CEACAM6, which makes interactions between immunocytes and CEACAM6-positive tumor cells more likely through homophilic and heterophilic intercellular adhesion. Thus, most tumor cells are suppressed or killed, resulting in a better OS in patients with early-stage GC (Fig. 6A). With tumor development, the body’s immune system is weakened. The number of immunocytes significantly reduces, while the expression of CEACAM6 increases. Thus, tumor cell migration, anti-apoptosis, and angiogenesis are markedly increased in advanced-stage GC. Consequently, the tumor suppressing effects of the immune response are weakened, while the tumor promoting effects of CEACAM6 significantly increase, resulting in poor OS in patients with advanced-stage GC (Fig. 6B).

## Discussion

OS is a very important factor for assessing the prognosis of cancer patients^23–25^. In this study, we used Kaplan-Meier survival estimates to evaluate the role of CEACAM6 and CEA expression in predicting the OS of patients with GC. We observed that CEACAMs played a somewhat contradictory role in predicting OS in patients with GC, with high CEACAM6 and CEA expression associated with better OS in early-stage GC, but poor OS in advanced-stage GC. This paradoxical finding was unexpected. Since CEACAM6 promotes GC metastasis and angiogenesis, while inhibiting GC differentiation, high CEACAM expression was expected to result in a poor OS in patients with GC^20, 21, 26, 27^. The reason for our observation is unclear. Potential explanations are discussed below.

We observed that CEACAM6 expression has no effect on the OS of patients with GC despite the role of CEACAM6 in promoting cancer metastasis. However, in advanced-stage GC, high CEACAM6 expression is associated with a poor OS. CEACAM6 expression increased with GC progression and was associated with cell structure disorder, suggesting CEACAM6 inhibits cell differentiation in GC, as well as, in colon cancer^27^. The migrating CEACAM6-positive tumor cells were not detected in adjacent tissues in early-stage GC, they were detected in vascular and interstitial tissues in advanced-stage GC. Scattered CEACAM6-positive tumor cells and tumor mass in adjacent tissues may be the consequence of invasion of strongly CEACAM6-positive tumors. As time passes, scattered tumor cells may replace normal surrounding tissues. Moreover, we show that CEACAM6 overexpression also increases the resistance of GC cells to anoikis, which in turn enhances the metastasis and growth of CEACAM6-positive tumors. Our findings may contribute to a better understanding of why high CEACAM6 expression leads to poor OS in patients with advanced-stage and poorly differentiated GC.

A greater number of CD4- and CD8-positive cells were detected in early-stage GC tissues than in advanced-stage GC tissues, suggesting lymphocytes are dramatically reduced in advanced-stage GC, leading to poor OS. CEACAMs are widely expressed in immune cells^11, 16, 17^. CEACAM6-positive immune cells are more likely to interact with CEACAM6-positive tumors through homophilic and heterophilic intercellular adhesion, thus suppressing tumor development. The CEACAM6 vaccine induced T-lymphocyte and natural killer cell infiltration and inhibited tumor development in rat colon cancer^28^. In CEA transgenic mice, a lentiviral vector expressing human CEA induced both cellular and humoral immune responses, such as CD8-positive T-lymphocyte infiltration and antibody secretion, thus enhancing immunotherapy against CEA-positive tumors^29^. These observations may explain why high CEACAM6 expression is associated with better OS in patients with early-stage GC.

In addition, our findings showed that OS was better for patients with GC who had received adjuvant chemotherapy without 5-FU than for those with high CEACAM expression who had received adjuvant chemotherapy with 5-FU. This suggests that patients with GC with high levels of CEACAM expression are not sensitive to 5-FU. Moreover, CEACAM6 overexpression in GC cells inhibited apoptosis and increased resistance to 5-FU. Increased resistance to 5-FU may account for the poor OS in patients with GC who have high levels of CEACAM6 expression and have received adjuvant chemotherapy with 5-FU. Thus, the use of 5-FU to treat patients with GC who express high levels of CEACAM6 and CEA may be inefficient and counterproductive, leading to poor OS. Furthermore, gastric adenocarcinoma samples from online database were classified into intestinal type, diffuse type, and mixed type based on Lauren classification. Compared to low CEACAM6 expression, high CEACAM6 expression is associated with better OS in intestinal type (*P* < 0.05). However, there are no statistic difference on OS between high and low CEACAM6 expression in diffuse type gastric adenocarcinoma (*P* > 0.05), as well as no difference in mixed type (*P* > 0.05; Supplementary Fig. S3).

Although CEACAMs are membrane proteins that are overexpressed in cancer tissues^20^, they can be recognized by immune cells and monoclonal antibodies from plasma cells, thus inducing tumor cell apoptosis and suppressing tumor development. CEACAM6 promotes tumor growth and metastasis in nude mice even though they are immunodeficient^20, 26^. Although CEACAM6 promotes tumor cell migration, invasion, and anti-apoptosis *in vitro*, the situation is different *in vivo* because of the presence of an immune system. In early-stage GC, the body’s immune response is strong enough to kill tumor cells despite the anti-apoptotic effect of CEACAM6, resulting in better OS. However, advanced-stage GC is characterized by a marked reduction in the number of immune cells, serious nutritional deficiency, and microenvironmental disorder, with a concomitant increase in CEACAM6 expression. Consequently, the body’s immune response weakens and can no longer resist the tumor promoting effects induced by the overexpression of CEACAM6, leading to poor OS. Combination immunotherapy that involves a tumor-antigen-targeting antibody, recombinant interleukin-2, an anti-programmed death-1 antibody, and a powerful T-cell vaccine is capable of eradicating large established tumors in mice^30^. The CEACAM6 antibody and vaccine significantly inhibited tumor development^28, 31, 32^. These observations suggest that combination immunotherapy and antibodies against CEACAM6 and CEA may be effective treatments for GC.

Tumor marker CEA has been used for several decades to predict the prognosis of tumor patients. In this study, the OS of patients with different stages of GC was clearly predicted based on CEACAM6 expression (Fig. 1A–H and Table 1), rather than CEA expression (Supplementary Fig. S1A–E and Table S1). Furthermore, CEACAM6 expression has been shown to be more abundant than CEA expression in breast, pancreatic, lung, and colon cancer^33^. In colorectal cancer, CEACAM6 overexpression independently predicted poor OS and disease-free survival, whereas CEA was not significantly related to these outcomes^34^. In addition, CEACAM6 is also a marker for early GC^35^. Thus, CEACAM6 was better than CEA as a tumor marker for predicting OS in patients with GC. However, there are some limitations to this study, including its retrospective design. Large-scale, prospective, multicenter, randomized controlled trials are needed to confirm our findings, which will in turn, guide antitumor treatment regimens.

In conclusion, high CEACAM6 expression results in better OS in early-stage GC, but poor OS in advanced-stage GC when the body’s immune response is weakened. CEACAM6 may be a better tumor marker than CEA for predicting the OS of patients with GC. In addition, CEACAM6 increases tumor cell resistance to 5-FU. Therefore, combination immunotherapy against CEACAM6, to enhance the body’s immune system, and other non-5-FU-based chemotherapy regimens should be considered for the treatment of early- and advanced-stage GC.

## Methods

### Statement

In this study, all methods were carried out in according to the guidelines and regulations. All experiment protocols about the cells and human tissues were approved by Shanghai Key Laboratory of Gastric Neoplasms, Shanghai Institute of Digestive Surgery, Ruijin Hospital, Shanghai Jiao Tong University School of Medicine. This study was approved by the Ethics Committee of Shanghai Ruijin Hospital, Shanghai Jiao Tong University School of Medicine.

### Tissue samples

Gastric tumor and adjacent non-tumorous tissue were obtained from 160 patients with GC who underwent curative surgery at Ruijin Hospital. None of the patients had received radiotherapy or chemotherapy prior to surgery. All patients were fully informed of the experimental procedures and informed general consent was obtained from all participants.

### Cell culture and materials

The human GC cell lines SGC-7901 and MKN-45 were purchased from Shanghai Institutes for Biological Sciences, Chinese Academy of Sciences. Cells were cultured at 37 °C in 5% CO_2_ and saturation humidity in RPMI-1640 medium containing 10% fetal bovine serum.

### Vector construction

The method CEACAM6 overexpressing vector constructed was consist with the previous^21^. We assembled a pIRES2-eGFP-CEACAM6 construct by inserting CEACAM6 cDNA into pIRES2-eGFP vector. Moreover, vectors were transfected into GC cells using Lipofectamine 2000 (Invitrogen, Carlsbad, USA) in according to the manufacturer’s protocol.

### Apoptosis assay

The method was consistent with the previous^36^. SGC-7901-CEACAM6, MN-45-CEACAM6, and their control cells were plated in 6-well plates treated with or without 5-Fu for 24 h. Cells were collected and examined by Apoptosis Detection Kit (BD Pharmingen). Apoptosis assay was monitored by flow cytometry.

### Anoikis assay

Anoikis of GC cells was performed using the CytoSelect 24-well Anoikis Assay Kit (Cell Biolabs; San Diego, CA, USA) according to the manufacturer’s instructions.

### Immunohistochemistry

The method was consistent with the previous^26^. Immunohistochemical staining of sections was performed according to the DAKO protocol, using mouse anti-CEACAM6 (1:400; Abcam). Immunohistochemistry stain score = positive cell score × staining intensity score. The percentage of positive cells was scored as follows: 0 (10%), 1 (10–25%), 2 (26–50%), 3 (51–75%) and 4 (>75%). Immunohistochemical staining intensity was graded as follows: 0 (no staining), 1 (weak staining), 2 (brown staining), and 3 (dark brown staining). Total scores of ≥ 3 were defined as positive to simplify data analysis.

### Kaplan Meier plotter

The Kaplan-Meier plotter (http://www.kmplot.com/analysis/) utilizes 1,065 GC cases (mean follow-up, 33 months) to assess the effect of genes on survival. Gene expression and relapse-free and OS data were downloaded from the Gene Expression Omnibus (Affymetrix microarrays only), European Genome-phenome Archive, and The Cancer Genome Atlas. The database is handled by a PostgreSQL server that integrates samples from three major cancer research centers in Berlin, Bethesda, and Melbourne, as well as, publicly available datasets with available follow-up data. Clinical information was collected from the Gene Expression Omnibus for 882 GC patients, including stage, the extent of tumor differentiation, and the manner of treatment. To analyze the prognostic value of CEACAM6, “CEACAM6” (Affymetrix ID: 211657_at) was entered into the gene symbol field and Kaplan-Meier plots were drawn. Patients were stratified into high and low expressing groups, which were compared using the log-rank test and hazard ratios with 95.0% confidence intervals. Each database is updated biannually.

### Data availability

The datasets generated during and/or analysed during the current study are available from the corresponding author on reasonable request.

### Statistical analyses

“CEACAM6” and “CEA” were entered into the web server and the corresponding OS data for GC patients was obtained. Log-rank tests were used to evaluate statistical differences in OS between the high and low expressing groups. Student’s *t*-tests were used to examine differences in apoptosis, anoikis, and CD4/8 staining scores between the two groups. All statistical analyses were conducted using Statistical Package for the Social Sciences for Windows (software version 19.0; IBM Corp., Armonk, NY, USA). A two-tailed *P < *0.05 was considered statistically significant.

## Electronic supplementary material

Supplementary Info
