# Supplementary material for: Dual role of carcinoembryonic antigen-related cell adhesion molecule 6 expression in predicting the overall survival of gastric cancer patients
Source: Sci Rep. 2017 Sep 7;7:10773. doi: 10.1038/s41598-017-11482-9 (PMC5589957; doi:10.1038/s41598-017-11482-9)
Supplement: Supplementary file 1 — Supplementary Info [file 41598_2017_11482_MOESM1_ESM.pdf]

Title: Dual role of carcinoembryonic antigen-related cell adhesion molecule 6 expression in predicting the overall survival of gastric cancer patients.

Authors: Mingde Zang[1,*], Lei Hu[1,*], Shu Cao[1], Zhiyuan Fan[1], Li Pang[1], Jianfang Li[1], Liping Su[1], Chen Li[1], Wentao Liu[1], Qinlong Gu[1], Zhenggang Zhu[1], Min Yan[1], Bingya Liu[1,†]

Affiliate: [1]Department of General Surgery, Shanghai Key Laboratory of Gastric Neoplasms, Shanghai Institute of Digestive Surgery, Ruijin Hospital, Shanghai Jiao Tong University School of Medicine, Shanghai 200025, People's Republic of China.

* These authors contributed equally to this work.

†**Corresponding author:** Bingya Liu; E-mail: **liubingya@sjtu.edu.cn**; Tel: 86-21-64670644; Fax: 86-21-64393909.

Supplementary figures

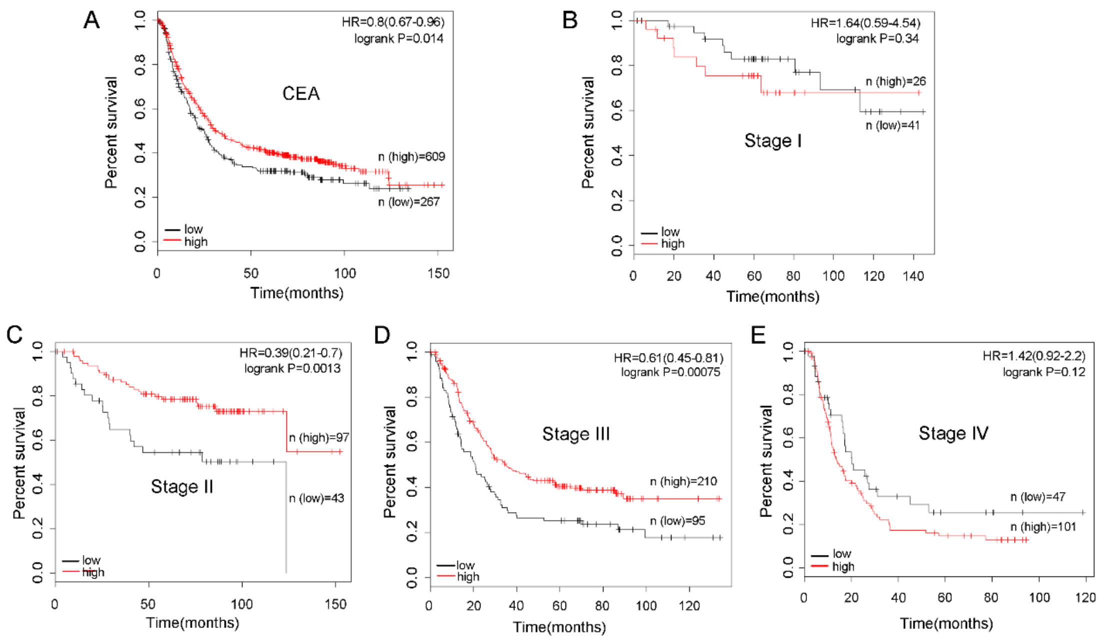

Supplementary figure S1. Role of CEA expression in predicting the overall survival in patients with gastric cancer. Kaplan-Meier curves of overall survival according to (**A**) CEA expression, (**B**) CEA expression in Stage I GC, (**C**) CEA expression in Stage II GC, (**D**) CEA expression in Stage III GC, and (**E**) CEA expression in Stage IV GC.

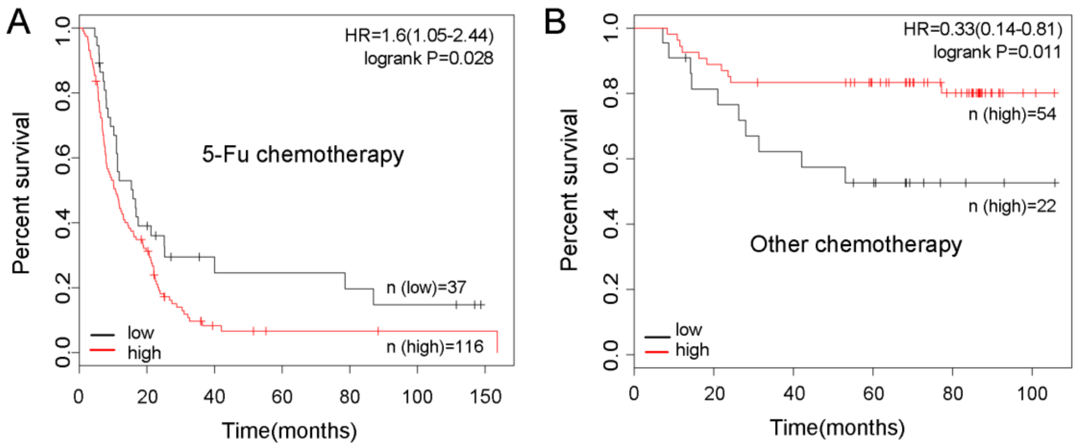

Supplementary figure S2. Role of CEA expression, and chemotherapy with 5-fluorouracil in predicting overall survival in patients with gastric cancer. Kaplan-Meier curves of OS according to CEA expression in patients who were treated with adjuvant chemotherapy (**A**) with and (**B**) without 5-fluorouracil.

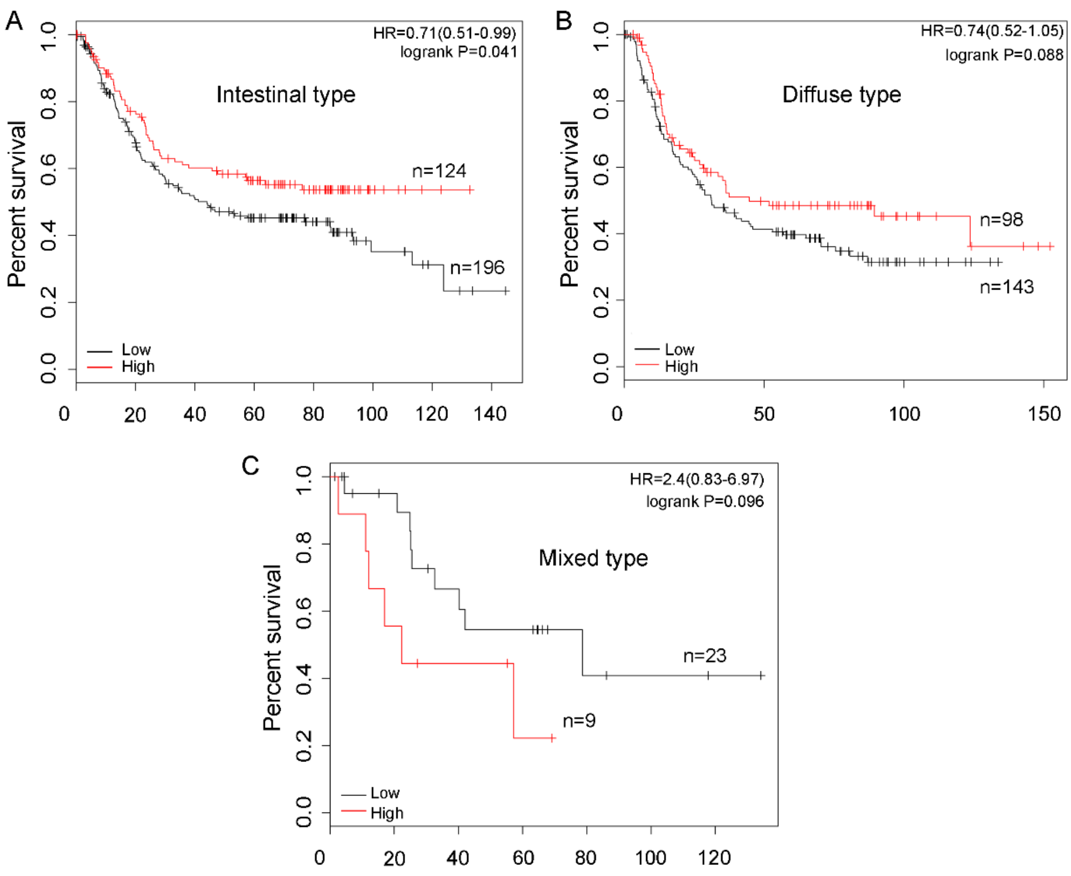

Supplementary figure S3. Role of CEACAM6 expression in predicting the overall survival in patients with gastric cancer. Kaplan-Meier curves of OS according to (**A**) CEACAM6 expression in intestinal-type GC, (**B**) CEACAM6 expression in diffuse-type GC, (**C**) CEACAM6 expression in mixed-type GC.

Supplementary table

Table S1. Association between CEA expression and overall survival of gastric cancer patients (n=876) (data from http://kmplot.com/analysis/)

Parameters		Cases	Median Survival(months)	HR(95%CI)	P value
All patients	Low	267	24.9	0.8 (0.67-0.96)	**0.014**
	High	609	31		
I	Low	41	93.2	1.64 (0.59-4.54)	0.34
	High	26	63.7		
II	Low	43	25.5	0.39 (0.21 -0.7)	**0.0013**
	High	97	85.8		
III	Low	95	20.9	0.61 (0.45-0.81)	**0.00075**
	High	210	35.1		
IV	Low	47	20.3	1.42 (0.92-2.2)	0.12
	High	101	13.73		
5-Fu chemotherapy	Low	37	15.9	1.6 (1.05-2.44)	**0.028**
	High	116	10.7		
No 5-Fu chemotherapy	Low	22	NA	0.33 (0.14-0.81)	**0.011**
	High	54	NA		

NA: Not Available. HR: Hazard Ratio.
